# Baltimore Journal of Medicine—A New Journal

**Published:** 1861-01

**Authors:** 


### A New Journal.

We are in receipt of the prospectus of a Bi-Monthly Medical Journal, to make its appearance in the present month, under the name of "*Baltimore Journal of Medicine*," and edited by Edward Warren, M.D., Professor of Materia Medica and Therapeutics in the University of Maryland.

It will be remembered that Dr. Warren, for a number of years, conducted, as editor, the *North Carolina Medical Journal*, at Edenton, N. C., with marked ability. He won for himself an enviable character during his service in Medical Journalism at his former location, and we bespeak for him, in his new field of action, the confidence and patronage which his talent and gentlemanly bearing so richly merit. Success, say we, to the *Baltimore Journal of Medicine*, and to Dr. Warren, whatever position he may be called to fill. This Bi-Monthly will contain not less than one hundred pages, at $3 per annum, *in advance;* if not paid until the receipt of the *second number*, $3.50.

☞ *The Nashville Medical Record* comes to us, after an absence of a month or two, with an entire change in its physical form and dimensions. In place of the usual octavo, it now assumes the quarto form, with 18 double column pages, resembling in these respects *The American Medical Times*. It is now edited by J. J. Abernathy, M. D., Thos. Maddin, M. D. and John H. Callender, M. D.—all of Shelby Medical College, Nashville, Tenn.

Success, say we, gentlemen, in your laudable endeavors to forward the great cause of Medical science.

"To *What affections of the lungs does Bronchitis give origin*," *A Prize Essay*, by Daniel Denison Slade, M.D., of Boston. A pamphlet of 65 pages with the above title has been received from the author. Every thing connected with diseases of the respiratory organs is interesting to the practicing physician. In the essay we have a discription of the symptoms of, and the changes produced in the bronchil and their secretions by bronchitis. Next is enumerated and described the diseases of the lungs which originate in bronchitis. These are collapsion, pneumonia, pleurisy. The results are set down as atrophy of the lungs, pulmonary concretions, emphysema, &c.

The essay is written in fair style, and is worth perusal.—Many important practical facts are correctly given.

We are in receipt of Peters, Harden & Co.'s Catalogue of "Fruit and ornamental trees &c." Downing Hill Nursery, Atlanta Ga. Their collection is not only of interest to the horticulturalist and the lover of ornamental shrubbery and flowers, but will be found to contain many valuable medicinal plants, both exotic and indigenous. Nothing is wanting on the part of the polite and accommodating superintendent, Mr. Robinson, to make visitors to the nursery pass the time pleasantly, and to give them an idea of the character and history of the various plants in the collection. We have frequently taken the opportunity of examining their plants, particularly those medicinal, and have been able to make considerable additions to the "Medicinal Garden" connected with the College, from this source.

